# Detection of Androgenic-Mutagenic Compounds and Potential Autochthonous Bacterial Communities during *In Situ* Bioremediation of Post-methanated Distillery Sludge

**DOI:** 10.3389/fmicb.2017.00887

**Published:** 2017-05-17

**Authors:** Ram Chandra, Vineet Kumar

**Affiliations:** Department of Environmental Microbiology, School for Environmental Sciences, Babasaheb Bhimrao Ambedkar UniversityLucknow, India

**Keywords:** distillery sludge, toxicity, β-sitosterol, *Bacillus* sp., *Enterococcus* sp., RFLP

## Abstract

Sugarcane-molasses-based post-methanated distillery waste is well known for its toxicity, causing adverse effects on aquatic flora and fauna. Here, it has been demonstrated that there is an abundant mixture of androgenic and mutagenic compounds both in distillery sludge and leachate. Gas chromatography-mass spectrometry (GC-MS) analysis showed dodecanoic acid, octadecanoic acid, *n*-pentadecanoic acid, hexadecanoic acid, β-sitosterol, stigmasterol, β-sitosterol trimethyl ether, heptacosane, dotriacontane, lanosta-8, 24-dien-3-one, 1-methylene-3-methyl butanol, 1-phenyl-1-propanol, 5-methyl-2-(1-methylethyl) cyclohexanol, and 2-ethylthio-10-hydroxy-9-methoxy-1,4 anthraquinone as major organic pollutants along with heavy metals (all mg kg^-1^): Fe (2403), Zn (210.15), Mn (126.30, Cu (73.62), Cr (21.825), Pb (16.33) and Ni (13.425). In a simultaneous analysis of bacterial communities using the restriction fragment length polymorphism (RFLP) method the dominance of *Bacillus* sp. followed by *Enterococcus* sp. as autochthonous bacterial communities growing in this extremely toxic environment was shown, indicating a primary community for bioremediation. A toxicity evaluation showed a reduction of toxicity in degraded samples of sludge and leachate, confirming the role of autochthonous bacterial communities in the bioremediation of distillery waste *in situ*.

## Introduction

Various forms of industrial waste are major sources of environmental pollution due to the release of several unidentified toxic pollutants. The majority of agro-based industries, i.e., distilleries, tanneries, and pulp paper are major sources of aquatic and soil pollution. Due to the release of huge amounts of waste water and sludge, sugarcane-molasses-based distilleries are among the most polluting industries in India. Distilleries release 12 to 15 l of spent wash per liter of alcohol produced. Currently, there are more than 319 distilleries in India, reflecting the magnitude of the problem due to the presence of various complex pollutants in post-methanated distillery effluent (PMDE) and post-methanated distillery sludge (PMDS). The detection and detoxification of distillery waste is a challenge for the safe disposal of effluent and sludge. Safe disposal of PMDS in the environment is of paramount importance currently due to the presence of various unidentified complex organic and inorganic pollutants (melanoidins, phenolics, and sulfur compounds as well as heavy metals) (Chandra and Kumar, 2017a). These complex pollutants in PMDS are generated during the process of the distillation of fermented molasses slurry and the subsequent methanogenesis of the spent wash. The toxicity of PMDS and PMDE to the terrestrial and aquatic environment are well documented due to the presence of heavy metals and organic compounds (Bharagava and Chandra, 2010). The seed germination (SG) test has indicated stunted stem growth and reduced root systems in *Phaseolus mungo* at higher concentrations of sludge-amended soil (Chandra et al., 2008).

Microbial communities are fundamental components of any ecosystem, playing a primary and critical role in the metabolism of organic matter to maintain biogeochemical cycles in various critical environments (Fuhrman, 2009). They are predominantly involved in the bioremediation of contaminated sites, and several microorganisms, which degrade a wide range of pollutants have been described (Loviso et al., 2015). A detailed knowledge of microbial communities at any polluted site not only reflects their relationships with pollutants, but also reflects information with respect to the bioremediation potential of microbes on specific pollutants. This may indicate a direction of the bio-stimulation or bio-augmentation for the restoration of any polluted ecosystem.

However, recent progress in molecular microbial ecology has shown that traditional culturing methods are insufficient to allow detailed analysis of the microbial diversity at any contaminated site. This is because only a small proportion of viable microorganisms from a sample are recovered by culturing techniques. So, to show the full extent of microbial diversity various molecular techniques have been applied (Zhongtang and Mohn, 2001). The study of pollutants and their influence on the microbial communities of WWTPs can provide useful information for solving the problem of fluctuation in WWTPs. The study has also demonstrated that different configurations of treatment plants influence the structures of microbial communities (Gich et al., 2000).

Some significant research has been carried out on the analysis of bacterial communities derived from the activated sludge of wastewater treatment processes and bioreactors, which was designed to evaluate the process performance of treatment plants for the biodegradation of hazardous chemicals (Whiteley and Bailey, 2000; Forney et al., 2001). For the assessment of the biodegradability of toxic compounds and the measurement of whole bacterial communities, substrate utilization has been reported using phospholipid fatty acid (PLFA) analysis and fluorescence *in situ* hybridization (FISH) techniques (Maharana and Patel, 2014). Rapid community fingerprinting by the polymerase chain reaction (PCR)-based denaturing gradient electrophoresis (DGGE) of 16S rDNA also indicated highly structured bacterial communities growing in treatment plants and different classes of bacteria were detected (γ*-Proteobacteria, Firmicutes*, etc.; Kapley et al., 2015). Some gene probes have also been used as a tool to detect the degradation of phenolic compounds in wastewater treatment systems (Purohit et al., 2003). A few studies have been published in the literature on microbial community indices using fatty acid or phospholipid analysis as a tool for the assessment of bioremediation of pollutants and eco-restoration of contaminated sites (Maharana and Patel, 2014). Detailed knowledge regarding organic pollutants and the growth of autochthonous bacterial communities at several complex polluted industrial waste sites is lacking. These recalcitrant pollutants persist in the environment and cause adverse effects on aquatic and soil ecosystems, and on flora and fauna, as well as on humans due to the presence of some unidentified mutagenic and androgenic compounds, which have, so far, not been isolated. So, the extraction of organic pollutants from PMDS using appropriate solvents would allow the extraction of the maximum number of toxic organic pollutants present in sludge. Their further detection by gas chromatography-mass spectrometry (GC-MS) will show the properties of the pollutants that challenge safe disposal, while simultaneously investigating the autochthonous bacterial communities present in disposed PMDS that mediate the bioremediation of toxic compounds *in situ*. Hence, an analysis of the growing autochthonous bacterial communities present in PMDS after 90 days of *in situ* bioremediation would not only provide information on the bacterial communities, it will also highlight a prerequisite step for the monitoring of polluting discharges from sugarcane-molasses-based distillery waste. Furthermore, the characterization of metabolic products will also provide important information about the environmental fate of these pollutants. The *in situ* bioremediation of organochlorine-pesticides-contaminated soil by using gene probes has been reported, but there is no knowledge of the chemical properties of pollutants present in complex industrial sludge discharge from sugarcane-molasses-based distillery waste after bio-methanogenesis. Furthermore, the autochthonous bacterial communities growing in sludge, which have potential capabilities for *in situ* and *ex situ* bioremediation remain unidentified.

In the present study, we have detected organic pollutants by GC-MS extracted with organic solvents to ensure the extraction of the majority of pollutants from disposed sludge. Energy dispersed spectroscopy (EDS) of sludge was also carried out for qualitative and quantitative analysis of heavy metals and of various salts. Potential autochthonous bacterial communities have also been detected using culture-independent methods, i.e., restriction fragment length polymorphism (RFLP) during *in situ* bioremediation to show the relationship between pollutants and the potential autochthonous bacterial communities responsible for biodegradation of PMDS. These findings will allow exploration of the potential autochthonous bacterial communities existing in the extreme environment of PMDS, which are capable of the bioremediation of inorganic and organic pollutants that are rich with phenolic and melanoidin compounds. Such bioremediation currently poses a challenge for the safe disposal of PMDS in the environment due to its complex structural and recalcitrant properties and its toxic effects on ecosystems.

## Materials and Methods

### Site Description, Sample Collection, and Preparation of Sludge Leachate

The fresh PMDS sample was collected in clean pre-sterilized polythene bags from the effluent treatment plant (ETP) of M/s Unnao Distillery and Breweries Limited, located in Unnao (26°320” N, 80°30′0”E) Uttar Pradesh, India. The plant has a capacity for 9000 kL of alcohol production and generates ∼800 tons of sludge annually (All India Distillers Association [AIDA], 2004). After 90 days of *in situ* bioremediation the degraded sludge sample was collected from the sludge dumping site of the distillery plant, which is located inside the premises. All the sludge samples collected were transported to the laboratory and used for the analysis of physicochemical parameters, detection of organic pollutants, detection of bacterial communities and preparation of leachate. The sludge leachate was prepared by stirring it in distilled water (1:1 w/v) for approximately 48 h and allowing the sludge suspension to stand still for 6 h in an Erlenmeyer flask. Clear supernatant was pumped out and filtered by passing through Whatman filter paper 42. Newly prepared sludge leachate was taken as 100%, with different concentrations prepared from it by adding distilled water to a final concentration of 1.0, 2.5, 5, and 10% for the analysis of various physicochemical parameters, detection of organic pollutants, phytotoxicty, and genotoxicity assays.

### Chemicals and Reagents

The organic solvents, *n*-hexane (C_6_H_14_), was procured from Merck (Merck KGaA, Darmstadt, Germany) and used for the extraction of organic compounds from distillery sludge. The following derivatizing reagents were obtained from Sigma-Aldrich (Saint Louis, MO, United States): pyridine, BSTFA [*N,O*-bis (trimethylsilyl) trifluoroacetamide] and TMCS (trimethylchlorosilane). All the chemicals and reagents used in this study were of HPLC grade.

### Physicochemical Analysis of Distillery Sludge and Sludge Leachate

The physicochemical parameters of fresh distillery sludge and sludge leachate samples, i.e., pH, electrical conductivity (EC), salinity, chloride, sodium, nitrate, ammonical nitrogen were estimated according to the method of Kalra and Maynard (1991). The pH and EC of sludge samples (1:1 sludge:water) and leachate were measured using an Orion meter (Model-960, Thermo Scientific, United States) and an Orion conductivity meter (Model-150, Thermo Scientific, United States), respectively. The heavy metals in the sludge and leachate samples were analyzed with standard methods (American Public Health Association [APHA], 2012) by atomic absorption spectrophotometry (AAS) (ZEEnit 700, Analytic Jena, Germany) after nitric acid-perchloric acid digestion method no. 3030H. Elemental analysis was performed for total C, N_2_, H_2_, and O_2_ by using an elemental analyzer (EuroVector EA 3000, University of AL-al-Bayt, Jordan). In addition, the SEM-EDS analysis of PMDS was performed using a scanning electron microscope (JEOL JSM-6490LV, Peabody, MA, United States). Operating conditions were: accelerating voltage 20 kV, probe current 45 nA and counting time 60 s. The physicochemical parameters were also analyzed in the degraded sample after 90 days of bioremediation of sludge *in situ*.

### Extraction and Identification of Various Organic Compounds from Fresh and Degraded Sludge

#### Solid Liquid Extraction of PMDS and Liquid–Liquid Extraction of Distillery Leachate

The fresh PMDS sample (5.0 g) was weighed and put into an Erlenmeyer flask (250 ml); 5 ml of *n*-hexane was added and it was mixed vigorously. Further, the samples were processed by vortex agitation (2 min), sonication (2 min on and 30 s off, ×3) and centrifuged (15 min 10,000 × *g*). In order to extract organic pollutants from distillery leachate, a fixed volume (10 ml) of sludge leachate was acidified with 35% (v/v) hydrochloric acid (HCl) and placed in a separating funnel (100 ml), after which an equal volume of *n*-hexane was added and the mixture was shaken continuously for 5 h with intermittent rests for liquid–liquid extraction. The extraction was repeated successively three times to complete the extraction of organic pollutants. Subsequently, the organic layer was collected from the sludge and leachate, dehydrated over anhydrous sodium sulfate (Na_2_SO_4_) and dried under a stream of N_2_ gas. A similar process was followed for the extraction of metabolic products from a degraded sludge sample after bioremediation *in situ*. The dry residue obtained was dissolved in 1.0 ml ethyl acetate and filtered through 0.22 μm syringe filters (Millipore Ltd., Bedford, MA, United States) and used for further Fourier transform-infrared spectrophotometry (FTIR) and GC-MS analysis.

#### Fourier Transform-Infrared Spectrophotometry

Fourier transform-infrared spectrophotometry analysis of purified extract was performed using a spectrophotometer (Nexus-890, Thermo Electron Co., Yokohama, Japan) in order to reveal the chemical nature of sludge and leachate. The purified samples were dispersed in spectral-grade KBr (Merck, Darmstadt, Germany) and made into pellets by applying 5–6 tons cm^-2^ of pressure for 10 min using a hydraulic pressure (Specac, United Kingdom) instrument. The spectrum was generated in the range of 400 to 4,000 cm^-1^ with a resolution of 4 cm^-1^ for all samples (fresh sludge, fresh sludge leachate, and degraded leachate).

#### Gas Chromatography-Mass Spectrometry (GC-MS) Analysis

In GC-MS analysis, the extracted PMDS and leachate samples were derivatized with trimethylsilyl (TMS) as described by Minuti et al. (2006). In this method, 50 μl pyridine and 80 μl TMS BSTFA, and TMCS were added to 300 μl samples. The mixture was heated at 70°C for 30 min, with periodic shaking to dissolve residues. An aliquot (2.0 μl) of silylated sample was automatically injected into a GC-MS (Thermo Scientific Trace GC Ultra Gas Chromatograph) equipped with a TriPlus auto sampler coupled to TSQ Quantum XLS triple quadrupole mass spectrometer (Thermo Scientific, Miami, FL, United States). Separation was carried out on a DB-5MS capillary column [30 m length × 0.25 μm I.D. × 0.25 mm film thickness of 5% phenyl and 95% methylpolysiloxane (v/v)]. The flow rate of carrier gas (He) was maintained at 1.1 ml/min, GC oven temperature was started at 65°C (held for 2 min), increased to 230°C at a rate of 6°C/min and finally reached 290°C (held for 20 min) at a rate of increase of 10°C/min (Chandra and Kumar, 2017b). Transfer line temperature and ion source temperature were kept at 290 and 220°C, respectively. The mass spectrometer was operated in the positive electron ionization (+EI) mode at an electron energy of 70 eV with a solvent delay of 7 min. Initially, to confirm the derivatization of organic compounds, full scanning mode was used in the mass range of 45–800 amu. Similar methods were also followed for other samples. The organic compounds were identified by comparing their mass spectra with that of the National Institute of Standards and Technology (NIST) library available with the instrument and by comparing the retention times with those of available standard compounds.

### Analysis of Uncultured Bacterial Communities Growing in PMDS

#### Genomic DNA Extraction and Purification

The total genomic DNA from degraded PMDS samples after 90 days of *in situ* bioremediation was extracted following the protocol of Berthelet et al. (1996). An aliquot (2.0 g) of sludge sample was mixed with 5.0 ml of 0.1 M sodium phosphate buffer (pH 8.0) in screw-capped polypropylene micro-centrifuge tubes (10 ml capacity) containing 5.0 g silica beads (0.1 mm diameter) followed by addition of 500 mM Tris-HCl (pH 8.0) and 10% sodium dodecyl sulfate (SDS). The tubes were shaken for 5 min at high speed on a Mini-Bead beater to lyse the cells. After lyses, the lysate was centrifuged (15,000 × *g*; 10 min; 4°C) in a microfuge and the supernatant obtained was mixed with double the volume of 7.5 M ammonium acetate and incubated on ice for 10 min. The samples were then centrifuged (15,000 × *g*; 10 min; 4°C) in a microfuge and aliquots of 300–500 μl supernatant were purified by centrifugation (1,000 × *g*; 2 min) through a spin column (Bangalore Genei, India), which was previously equilibrated and slurried with 20 mM potassium phosphate buffer (pH 7.4).

#### PCR Amplification of 16S rRNA Genes

The PCR amplification of 16S rDNA genes derived from degraded PMDS was performed with universal eubacterial primers (27F) 5′ AGAGTTTGATCMTGGCTCAG 3′ and (1492R) 5′ TACGGYTACCTTGTTACGACTT 3′ using a Thermocycler (Sure Cycler-8800, Agilent Technologies, United States). The reaction mixture contained 5 μl of template DNA (1 × PCR buffer, 10 mM of each: dNTP, 3.0 mM MgCl_2_, 10 pmol of primer and 2.5 U of *Taq* DNA polymerase. Bangalore Genei, India) in a final volume of 50 μl reaction mixture. As a negative control, reactions without DNA were carried out. The complete reaction mixture was overlaid with mineral oil and incubated in a thermal cycler (Sure Cycler-8800, Agilent Technologies, United States). The cycling program was as follows: initial denaturation at 94°C for 1 min, primer annealing at 55°C for 1 min, a final extension at 72°C for 3 min with an additional extension time of 10 min added to the final cycle, for a total of 35 cycles. The PCR products (amplicon) were electrophoresed through 1.2 % (w/v) agarose gel in 1× TAE buffer using a 1 Kb DNA ladder (Bangalore Genei, India) as molecular weight markers and visualized by staining with ethidium bromide. The amplified 16S rDNA gene products were gel purified by using a PCR-Clean-up kit (Bangalore Genei, India) and used for the clone library preparation.

#### Cloning of 16S rDNA PCR Products

For cloning, 10 μl purified PCR products were made blunt-ended by treatment with 10 U of the large (Klenow) fragment of DNA polymerase I, after which the 5′ end was phosphorylated with 10 U of T4 polynucleotide kinase (Promega, Madison, WI, United States). The reaction mixture, also contained 10 μl 10 × Klenow buffer [0.5 M Tris HCl (pH 7.5 at 25°C), with 10 mM MgCl_2_, 10 mM dithiothreitol, 0.5 mg bovine serum albumin per ml, 1 mM ATP, 200 μM DdATP, 200 μM dCTP, 200 μM dGTP and 200 μM dTTP also present]; the total volume was 100 μl and the prepared solution was incubated at 37°C for 1.0 h (Moyer et al., 1994). The blunt-ended PCR-amplified 10S rDNA gene products were again purified with Quiaex and ligated into a SmaI-digested dephosphorylated pUC18 vector (Promega, Madison, WI, United States). Competent *Escherichia coli* XL1 blue MRF′ cells (Stratagene) were transformed using the method described by Morrison (1977). The positive recombinants were screened for α-complementation with X-Gal (5-bromo-4-chloro-3-indoyl-β-D-galactopyranosides) as a substrate on solid agar medium plates supplemented with ampicillin (150 μg/ml).

#### 16S rDNA RFLP Analysis

Recombinant plasmids were isolated from overnight cultures by alkaline lysis. The cloned 16S rDNA gene fragments were then digested with the restriction enzymes, *Taq*I and *Sau*3AI, either separately or together (Merck Biosciences, Maharashtra, India). Restriction digestion was performed at 37°C for 2 h in a 50 μl reaction mixture containing 12 μl (∼200 ng) of PCR-amplified 16S rRNA gene product, 0.5 μl (5U) of restriction endonucleases (*Taq*I/*Sau*3AI; Merck Biosciences, Maharashtra, India), 5 μl of reaction buffer and 32.5 μl of autoclaved Milli-Q water. The restriction digestion process was repeated twice in order to establish the reproducibility of results. The resulting RFLP products were separated by gel electrophoresis in 1.8% (w/v) agarose gel and Tris-borate-EDTA buffer at 75 V for 5 h. Next, gels were stained with EtBr (1.0 μg/ml) and DNA bands were visualized using the Gel Documentation System (Syngene, United States). The DNA fragments were compared with a 100 and 500 bp DNA ladder (Merck Biosciences, Maharashtra, India) to determine molecular weights and sizes.

#### Sequencing of Cloned 16S rDNA PCR Fragments

Based on differences in the RFLP profiles generated, 10 bands generated by digestion of DSW by *Taq*I and *Sau*3AI were selected for sequence analysis. Selected bands were gel purified using gel extraction kits (Merck Biosciences, Maharashtra, India) and sequenced using the M13 forward (5′-GTAAAACGACGGCCAGT-3′) and reverse universal primers (5′-CAGGAAACAGCTATGAC-3′) and an ABI PRISM^®^BigDye^TM^ Terminator Cycle Sequencing Ready Reaction Kit (Applied Biosystems, Waltham, MA, United States). The samples were then sequenced using an automatic DNA sequencer (ABI PRISM^®^310 Genetic Analyzer, United States). The partial sequences obtained were subjected to BLAST analysis using the online option available at http://blast.ncbi.nlm.nih.gov/Blast.cgi (Altschul et al., 1997).

#### Phylogenetic Analysis and Nucleotide Sequence Accession Number

A phylogenetic tree was generated using MEGA-6.0 software (Tamura et al., 2013). All query sequences and other homologous sequences available online in the NCBI (National Centre for Biotechnology Information)^1^ nucleotide database were saved in a single FASTA file format after retrieval. Furthermore, all sequences were saved in one FASTA format file and then subjected to multiple sequence alignment using MEGA-6.0, which was subsequently used to reconstruct phylogenetic trees by the Neighbor-Joining method using the MEGA-6.0 Draw Tree tool (Larkin et al., 2007) with a bootstrap value of 1,000 replicates. Nucleotide sequences were deposited in the GenBank Nucleotide database under accession numbers from FJ227523 – FJ227532.

### Toxicity Assessment of Sludge

#### Phytotoxicity Assay

The toxicity of the fresh and *in situ*-degraded sludge leachate was assessed by measuring the phytotoxicity effect on the germination of seeds of *Phaseolus mungo* L. using the Petri dish method (Santal et al., 2011). Prior to preparation of various concentrations of distillery leachate, the pH was adjusted to 7.0 with 1 M NaOH. For the SG experiment, distillery leachate was applied at 1.0, 2.5, 5, and 10% (v/v). The surfaces of the seeds were sterilized with 0.1% HgCl_2_ for 2 min to remove any fungal contamination, after which they were subjected to repeated washings with sterilized distilled water. Subsequently, 10 seeds of *P. mungo* L. were placed separately in sterilized glass Petri dishes of uniform size lined with two Whatman No. 1 filter paper disks. Disks were then moistened with 10 ml tap water for controls and with the same volume of distillery leachate, after which they were incubated at 25°C for a period of two consecutive days. The bioassay was performed on three replicate samples. The percentage SG and the percentage phytotoxicity (RI) was calculated with the formula previously described (Oleszczuk et al., 2012):

$$\begin{array}{c} SG/RI=\frac{\left(A-B\right)}{A}\times 100 \end{array}$$

Where, *A* is mean SG and root length in controls, *B* is mean SG and root length in test experiments.

Other SG parameters such as the germination index (GI), relative percentage toxicity, percentage phytotoxicity, and the stress tolerance index were calculated using the formula described by David Noel and Rajan (2015).

#### Genotoxicity Assay

The root tip cells of onion, *Allium cepa*, were used to test the genotoxic effects of fresh and degraded sludge leachate. The test was carried out as described by Fiskesjo (1985). The onion bulbs were previously germinated in tap water at room temperature. When the seeds reached about 2.0 cm in length, they were transferred to test tubes containing different concentrations of distillery sludge leachate (1%, 2.5%, 5% and 10%), while one tube with distilled water was used as a control. After 24 h of treatment, the root tips were collected and fixed in Carnoy’s fluid (ethanol-glacial acetic acid 3:1; v/v) for 24 h at 4°C, in order to arrest mitosis (Fiskesjo, 1993, 1997). Afterward, they were washed with distilled water and placed in 70% (v/v) ethanol for 24 h at room temperature. The root tips of bulbs were hydrolyzed with 1 M HCl at 60°C for 4–5 min to dissolve cell walls. After hydrolysis, the roots were washed with distilled water and approximately 1–2 mm of root tips were cut off and processed for staining with hematoxylin. Five slides were prepared for each concentration and for the control, out of which five (at 500 cells per slide) were analyzed with a light microscope (Leica Microsystem, Germany; ×1000). A total of 2500 cells were evaluated for each concentration. In the analysis, the following chromosomal and nuclear aberrations were considered: chromosome adherence, c-mitosis, chromosome bridges, vagrant chromosomes, disordered metaphase and anaphase, lagging chromosomes, multipolarity, and polyploidy. Mitotic index (MI) and mitotic inhibition were also an indication of cytotoxicity. In this study, MI and mitotic inhibition % was determined with the following formula, as described by Fiskesjo (1997):

$$\begin{array}{c} \mathrm{Mitotic}\;\mathrm{index}\;\left(\%\right)=\frac{\mathrm{Total}\;\mathrm{number}\;\mathrm{of}\;\mathrm{cells}\;\mathrm{in}\;\mathrm{division}}{\mathrm{Total}\;\mathrm{cell}\;\mathrm{number}\;\mathrm{of}\;\mathrm{cells}\;\mathrm{observed}}\times 100 \end{array}$$

$$\begin{array}{c} \mathrm{Mitotic}\;\mathrm{index}\;\left(\%\right)=\frac{Mitotic index in Control group - Mitotic index in test group}{Mitotic index in control group}\times 100 \end{array}$$

## Results

### Physicochemical Analysis of the Distillery Sludge and Leachate

The physicochemical properties of PMDS and its leachate are shown in **Table 1**. The valuehowed high concentrations of ions, e.g., Na^+^, Cl^-^, NO_3_^-^, and NH_4_^+^ (**Table 1**). It also had a high content of various heavy metals, where the highest content was noted for Fe followed by Mn, Zn, Cu, Cr, Pb, Ni, and Cd. Leachate analysis also demonstrated high values for pH and EC, BOD, COD, TDS, and various heavy metals (**Table 1**). This indicated the high leaching properties of the pollutants present in PMDS. However, the leachate obtained after 90 days of *in situ* bioremediation of PMDS had lower values of various physiochemical parameters (**Table 1**). This indicates that the biodegradation of various organic and inorganic content by autochthonous bacterial communities growing in PMDS had occurred. The SEM-EDS analysis of PMDS also showed heterogenous morphology (See Supplementary Figure 1), and its elemental composition included C (24.10%), O (50.08), Si (8.67%), Al (5.22%), and Fe (4.27%) as the main elements (See Supplementary Table 1).

**Table 1:** Physico-chemical analysis of disposed distillery sludge and leachate.

S. No.	Physico-chemical Parameters	Distillery sludgel	Distillery sludge leachate	Sludge leachate after *in situ* remediation	% Reduction ofsludge leachate
1	pH	8.00 @ 0.21 l	7.99 @ 0.10	7.114 @ 0.21ˆa	10.96
2	EC	4.1 @ 2.11 l	3.9 @ 1.14	2.4 @ 1.34ˆa	38.46
3	Sodium (Na^+^)	56 @ 1.31 l	55.108 @ 0.97	13.352 @ 0.80ˆa	75.77
4	Chloride (Cl^-^)	1825 @ 0.10 l	7034.6. @ 0.89	6878.674 @ 129.45ˆns	83.14
5	Nitrate (NO_3_^-^)	110 @ 3.14 l	109.916 @ 1.30	46.295 @ 0.90ˆa	57.88
6	Ammonical nitrogen (NH_4_^+^)	190 @ 1.24 l	187.794 @ 0.94	85.594 @ 0.71ˆa	54.42
7	TDS	–	16550.4 @ 2.07	2406 @ 89.19ˆa	85.56
8	BOD	–	11050.2 @ 2.28	2555.698 @ 1.78ˆa	76.87
9	COD	–	22421 @ 1.58	4512.934 @ 1.90ˆa	79.87
10	Total organic carbon	17.318 @ 0.21 l	16.404 @ 0.28	7.407 @ 1.09ˆa	66.48
11	Total nitrogen	2.463 @ 0.01 l	2.084 @ 0.04	1.982 @ 0.05ˆc	57.29
12	Total hydrogen	4.013 @ 0.04 l	3.668 @ 0.38	2.93 @ 0.05ˆc	69.41
13	Total oxygen	36.251 @ 1.11 l	35.408 @ 0.58	26.039 @ 3.52ˆb	9.42
14	Trace elements	l			
a	Iron (Fe)	2403 @ 3.11 l	1401.22 @ 1.86	852.528 @ 2.05ˆa	39.15
b	Zinc (Zn)	210.15 @ 2.14 l	95.273 @ 0.68	21.463 @ 1.67ˆa	77.47
c	Copper (Cu)	73.62 @ 1.14 l	62.928 @ 1.20	11.526 @ 1.02ˆa	81.68
d	Chromium (Cr)	21.825 @ 0.41 l	18.447 @ 0.60	9.140 @ 0.49ˆa	50.45
e	Cadmium (Cd)	1.440 @ 0.12 l	1.166 @ 0.15	0.94 @ 0.05ˆc	19.38
f	Manganese (Mn)	126.30 @ 0.94 l	94.602 @ 1.13	45.105 @ 0.72ˆa	52.32
g	Nickel (Ni)	13.425 @ 0.21 l	8.302 @ 0.31	2.250 @ 0.20ˆa	72.89
h	Lead (Pb)	16.33 @ 1.11 l	14.311 @ 1.65	7.222 @ 0.38ˆa	49.53

*All values are mean of three replicate ± SD and presented in mg kg^-*1*^ and ml L^*-1*^ for distillery sludge and distillery leachate, respectively, except electrical conductivity (μS cm^*-1*^), total carbon, total nitrogen, total hydrogen, and total oxygen (%). EC, electric conductivity; BOD, biological oxygen demand; COD, chemical oxygen demand; TDS, total dissolved solids; *t*-test (two-tailed as compared to fresh distillery leachate). ^a^Highly significant at *p* < 0.001. ^b^Significant at *p* < 0.01. ^c^Less significant at *p* < 0.05. ^ns^Non significant at *p* > 0.05.*

### Characterization of Organic Compounds

#### FTIR Analysis

**FIGURE 1:**
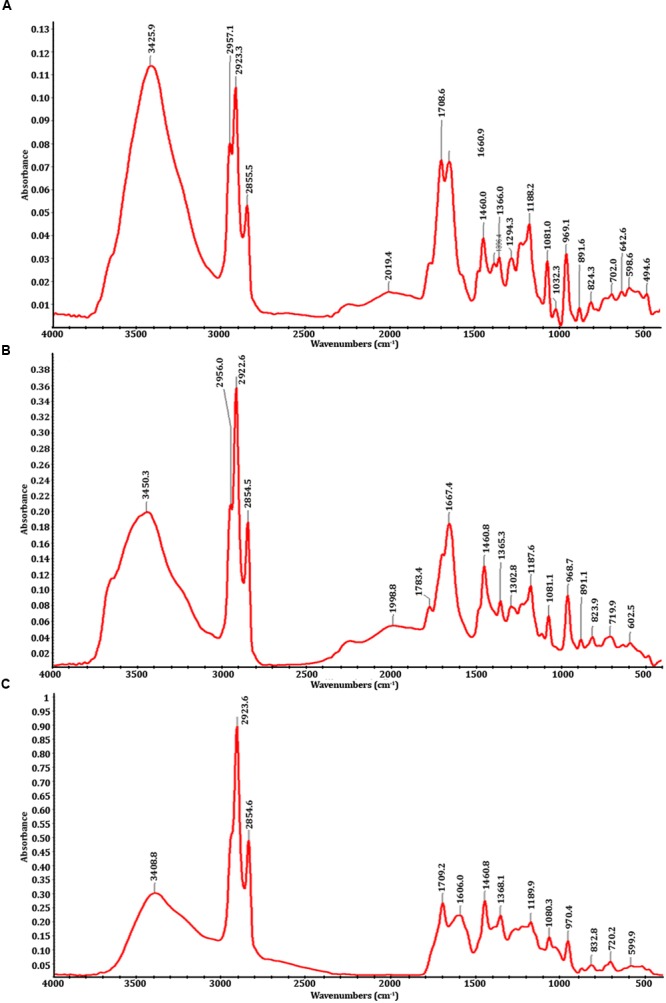
**FTIR spectra; (A)** fresh distillery sludge, **(B)** fresh distillery sludge leachate, **(C)** biodegraded distillery sludge leachate after 90 days of *in situ* bioremediation.

Fourier transform-infrared spectrophotometry analysis showed that there are significant differences between the spectra of PMDS, fresh sludge leachate and degraded sludge leachate obtained after *in situ* bioremediation by potential autochthonous bacterial communities (**Figure 1**). The infrared spectra (500–4000 cm^-1^) of distillery sludge were similar to the spectra obtained with distillery leachate. The FTIR spectra were analyzed based on the peak in control sludge, sludge leachate solution and in the treated samples, indicating the presence of different chemical bonds and various organic pollutants. The control region, as well as the degraded leachate at between 3200 and 3600 cm^-1^ represented broad peaks of stretching vibration indicative of O-H of COOH and the N-H stretching of amides. The peak values of 3425.9, 3450.3, and 3408.8 represented the O-H vibrational stretching present in acids, alcohols, and phenols. The region between 2800 and 3000 cm^-1^ exhibited C-H stretching due to the presence of -CH_3_ in hydrocarbon chains (de Oliveira Silva et al., 2012; Kadam et al., 2013). The controls and degraded samples showed peaks at 2854.5, 2854.6, 2855.5, 2922.6, 2923.3, 2923.5, 2956.0, and 2957.1 cm^-1^ of the C-H asymmetric stretching vibration of -CH, -CH_2,_ and -CH_3_ functional groups. In untreated distillery sludge and leachate, the broad stretching adsorption band peaks at 2019.4 and 1998.8 cm^-1^ could be assigned to C=C stretching vibrations. The band peak at 1081.0, 1081.1, and 1080.3 cm^-1^ was attributable to an O-H stretching vibration of the COOH band present in acids. A very strong band at 1,660.9, 1,667.4, 1,708.6, 1,709.0, 1,783.4, and 1,606 cm^-1^ was indicative of the C=O carbonyl stretching of secondary amides (Narain et al., 2012; Yang et al., 2015). There is a medium band at 1,396.4, 1,368.1, 1,365.3, 1,302.8 cm^-1^, which represents the C-H deformation of a -CH_2_, or CH_3_ group, while a band at 1,032.3 cm^-1^ indicates the presence of C-O stretching in polysaccharides. The spectrum region between 1,100 and 1,200 cm^-1^ of controls and degraded samples showed peaks at 1,188.2, 1,187.1, and 1,189.9 cm^-1^, which represents the S=O stretching in sulfone groups, while a band at 969.1, 968.7, and 970.4 indicates the presence of the C-O stretching of polysaccharides or an Si-O-asymmetric stretch. The band at 891.6, 891.1, 832.8, 824.3, 823.9, and 832.8 cm^-1^ represents the hydrogen-bonded OH- deformation in carboxyl groups (Kadam et al., 2013). Some peaks were also detected below 700 cm^-1^ due to the presence of multiple functional groups/compounds such as sulfates, carbohydrates, alkyl halides, nitro groups, and C-S bonds (Yang et al., 2015). The peaks at 702.0, 719.9, and 720.2 cm^-1^ were usually weak and due to the C-H bending vibrations of methyl groups. The absorption bands at 642.6 and 602.5 cm^-1^ were indicative of the S-O stretching vibration of sulfate compounds. The region in between 500 and 600 cm^-1^ represents the stretching vibration peaks of C-Cl, indicating the presence of alkyl chlorides. However, the sharpness of all the peaks diminished over time with *in situ* bioremediation, indicating the degradation of distillery leachate molecules into smaller ones and an overall decrease in organic compounds.

#### GC-MS Analysis

**FIGURE 2:**
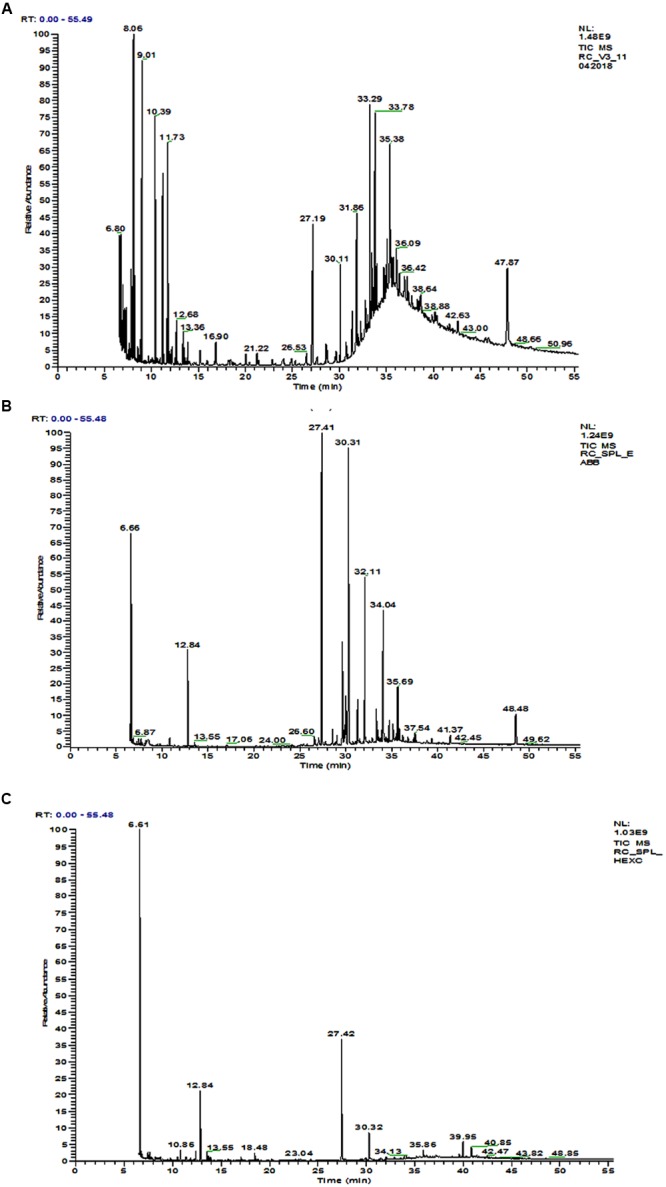
**GC-MS chromatogram of organic compounds extracted with *n*-hexane from; (A)** fresh distillery sludge, **(B)** fresh distillery leachate, **(C)** distillery leachate after 90 days of *in situ* bioremediation.

The GC-MS chromatogram of compounds extracted from PMDS and leachate with n-hexane is shown in **Figure 2** and organic compounds have been identified in detail at various retention times based upon mass to charge ratios (m/z). The results show that fresh PMDS and leachate contained large numbers of organic compounds as identified by GC-MS (**Table 2**). The major compounds were identified as saturated fatty acids (propanoic acid, dodecanoic acid, tetradecanoic acid, *n*-pentadecanoic acid, octadecanoic acid, and hexadecanoic acid). Other compounds were also identified as stigmasta-5, 22-dien-3-ol(3 β,22E), stigmasterol and β-sitosterol. Other compounds such as 1-propanol, 3-(octadecycloxy), D-lactic acid, TMS ether, TMS ester, 2-methyl-4-keto-pentan-2-ol, 1-methylene-3-methyl-butanol, benzene, 1,3-bis (1,1-dimethylethyl), phosphoric acid, 1-phenyl 1-propanol, 2-isoropyl-5-methyl-1-heptanol, 5-methyl-2-(1-methylethyl) cyclohexanol, 2-ethylthio-10-hydroxy-9-methoxy-1,4 anthraquinone, tert-hexadecanethiol and 2,6,10,14,18,22-tetracosahexane 2,6,10,18,19,23-hexamethyl were detected in sludge as well as sludge leachate samples.

**Table 2:** Identified organic compounds by GC-MS present in *n*-hexane extract of distillery sludge and leachate.

S. No.	RT	Name of compound	DS	DSLC	DSLD
1	7.33	1-Propanol, 3-(octadecycloxy)	–	+	+
2	8.15	2-Methyl-4-keto pentan-2-OL	+	+	–
3	8.16	D-Lactic acid, TMS ether, TMS ester	+	+	+
5	10.41	Trisiloxane, 1,1,1,5,5,5-hexamethyl-3,3-bis[(trimethylsilyl)oxy]-(CAS)	–	+	–
6	11.13	1-Methylene-3-methyl-butanol	+	–	–
7	12.46	Benzene, 1,3-bis(1,1-dimethylethyl)	+	+	–
8	12.86	Phosphoric acid	+	+	–
9	12.68	3,7-Dioxa-2,8-disilanonane, 2,2,8,8-tetramethyl-5-[(TMS)oxy	–	–	+
10	13.55	Docosanoic acid, docosyl ester	–	+	+
11	13.74	2-Isoropyl-5-methyl-1-heptanol	+	–	
12	14.84	1-Phenyl-1-propanol	+	+	–
13	15.70	Tetradecane	+	–	–
14	16.69	Tert-Hexadecanethiol	–	–	+
15	17.23	Decane, 2,3,5,8-tetramethyl	+	+	–
16	17.50	Propanoic acid	+	+	–
17	18.47	Benzoic acid, 3,5-bis(1,1-dimethylethyl)-4-hydroxy ethyl ester	–	–	+
18	19.12	1-Dodecanol	+	–	–
19	19.73	Docosane	+	+	–
20	20.73	Dodecanoic acid	+	–	–
21	21.62	Heptacosane	+	+	–
22	22.13	Dotriacontane	+	+	–
23	22.63	1-Hexadecanol, 2-methyl	–	–	+
24	23.01	Tert-Hexadecanethiol	+	–	–
25	24.24	Tetradecanoic acid	+	+	–
26	25.28	*n*-Pentadecanoic acid	+	+	–
27	27.15	Hexadecanoic acid, TMS ester	+	+	–
28	28.63	Palmidrol	–	–	+
29	30.34	Octadecanoic acid	+	+	–
30	31.85	Hexanedioic acid, bis(2-ethylhexyl ester)	–	+	+
31	34.71	Quercetin 7,3’,4’-trimethoxy	–		+
32	35.08	1H-Purin-6-amine, [(fluorophenyl)methyl	–	+	+
33	35.09	2-Monostearin TMS ether	–	–	+
34	35.91	2,6,10,14,18,22-Tetracosahexane 2,6,10,18,19,23-hexamethyl	+	+	–
35	37.41	Butyl 11-eicosenoate	–	–	+
36	41.33	Stigmasta-5,22-dien-3-ol(3β,22E)	+	+	–
37	41.55	Stigmasterol	+	+	–
38	42.14	Lanosta-8, 24 dien-3-one	+	+	–
39	42.61	1-Monolinoleoylglycerol TMS ether	–	–	+
40	42.40	Spirostan-3-one (5α, 20β, 25R)	+	–	+
41	42.66	β-Sitosterol trimethyl ether	+	+	–

*DS, distillery sludge; DSLC, distillery sludge leachate control; DSLD, distillery sludge leachate degraded after 90 days *in situ* bioremediation.*

However, in the GC-MS chromatogram of the n-hexane extract obtained from the degraded sludge sample after 90 days of *in situ* bioremediation the disappearance of several peaks and generation of some new peaks was clear. This indicated the degradation of major toxic organic compounds had occurred (mutagens, carcinogens, and environmental endocrine disrupters) along with the simultaneous biotransformation of some new compounds (**Figure 2** and **Table 2**).

### Characterization of Bacterial Communities Using 16S rRNA Gene Analysis

**FIGURE 3:**
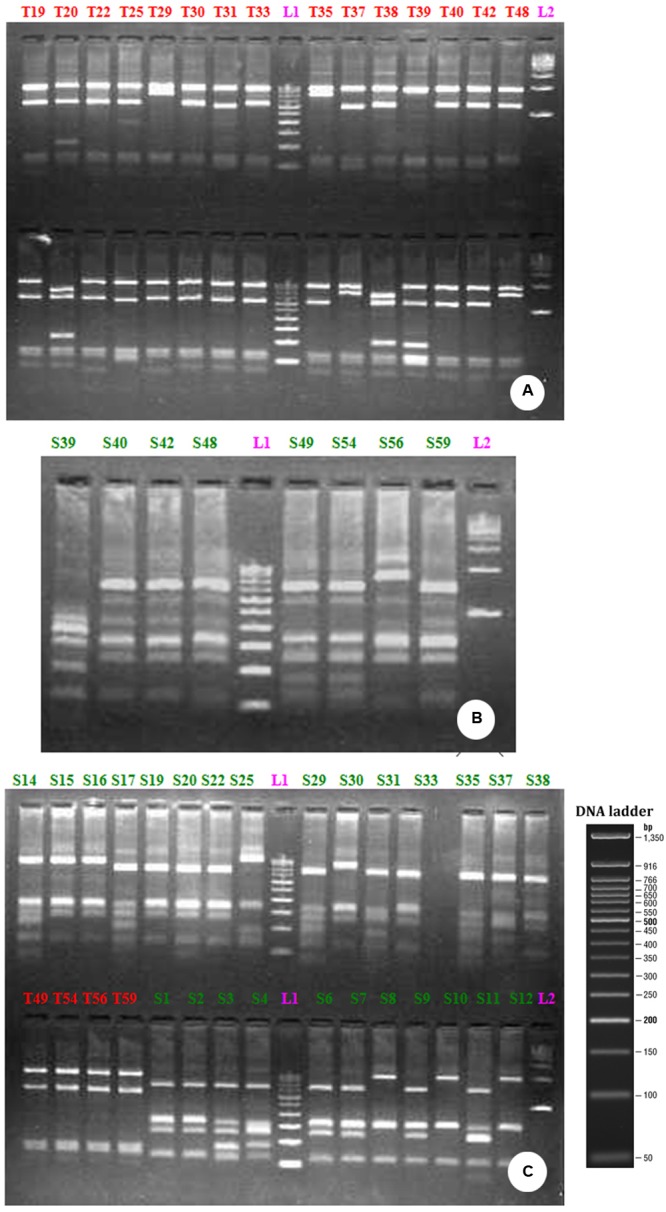
**RFLP profile generated by the restriction digestion of 16S rRNA gene derived from bacterial community growing on distillery sludge using *Taq* 1 (A)** *Sau*3AI **(B)** and *Taq* 1 and *Sau*3AI restriction endonuclease together **(C)**. The above picture shows the clones, labeled from T1-T48 **(A)** S39-S59 **(B)** T49-T59 are PCR positive products **(C)** L1-100 bp ladder; L2-500 bp ladder.

The restriction digestion of the 16S rDNA genes derived from the bulk DNA from the uncultured bacterial community growing in PMDS with *Taq*1 and *Sau*3A1 restriction enzymes demonstrated the presence of 10 clones of uncultured bacterial species (**Figure 3**) Specifically, fragments of 1503, 1496, 1515, 1519, 1515, 1521, 1515, 1500, and 1513 bp were observed. RFLP analysis indicated that clones of uncultured *Bacillus* sp. (9) were dominant in degraded PMDS followed by clones of *Enterococcus* sp. (1). On the basis of phylogenetic relationships, the dominant bacterial species identified belong to the phylum *Firmicutes* (See Supplementary Figure 2). Disposed PMDS might be a major source of microbial nutrients (nitrogen, phosphate, and carbon sources). The results indicate that the *Firmicutes* might have wide ranging metabolic capabilities, such that they are able to utilize various fatty acids, sugars and organic compounds as a sole carbon source. This ability would create a specific niche for these autochthonous bacterial communities, which may allow *in situ* bioremediation of pollutants.

### Phytotoxicity Assay

**FIGURE 4:**
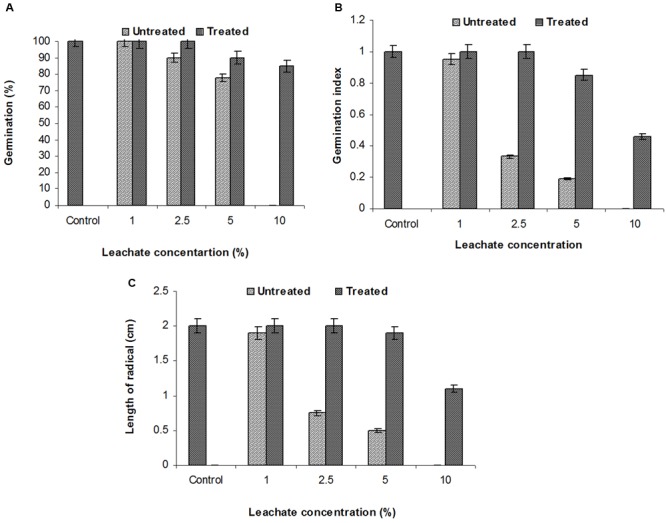
**Effects of various concentrations of sludge leachate on *Phaseolus mungo* L. vs. control (tap water); (A)** percent germination, **(B)** germination index, **(C)** radical length.

The SG test of green gram (*P. mungo* L.) showed inhibitory effects of fresh and degraded sludge leachate at different concentrations in terms of SG and growth parameters of seedlings (**Figure 4** and **Table 3**). Seeds germinated at rate of 100% with a 1% (v/v) concentration of fresh leachate, as the concentration increased the percent germination decreased, with 2.5 and 5% (v/v) resulting in 90 and 78% germination, respectively. There was no SG at a concentration of 10% (v/v) fresh sludge leachate after 24 h. However, in degraded sludge leachate 85% germination was recorded in up to a 10% (v/v) concentration, which was higher than with untreated leachate. With respect to the seedling growth (radical length), the radical length of seeds exposed to fresh (control) and degraded sludge leachate varied from 1.9 to 0.50 cm and 2.0 to 1.1 cm, respectively. When the seeds had been exposed to 10% (v/v) untreated leachate they showed no root development, but after treatment seeds showed development of a radical (1.1 cm). Inhibition of radical length was considered to be the first evidence of an effect of organic pollutants and of metal toxicity in plants. The germination percentage and the radical length were combined to give a comprehensive interpretation of leachate toxicity in terms of the GI. The GI values of untreated and treated leachate ranged between 0.19 to 0.95 and 1.0 to 0.46, respectively. The GI decreased with increasing concentrations of leachate. The maximum relative toxicity of control and degraded sludge leachate was noted as 22% at a concentration of 5% (v/v) and 15% at an 85% (v/v) concentration, respectively. The control sludge leachate showed greater relative toxicity. The percentage phytotoxicity analysis of the leachate obtained from controls revealed that phytotoxicity increased with increases in leachate concentrations, but in degraded leachate it gradually decreased. However, the extent of toxicity was found to be higher [75% at a 5% (v/v) concentration] of fresh sludge (control) leachate, while at the same concentration only 5% toxicity was noted in the degraded sludge leachate. The stress tolerance index of seedlings was at a minimum at a 5% (v/v) concentration of treated leachate. These results clearly indicate that the toxicity of sludge leachate was reduced significantly after *in situ* bioremediation.

**Table 3:** Effect of different concentration of distillery sludge leachate on seedling growth of *Phaseolus mungo* L.

Concentration (%)	Germination (%)		Relative toxicity (%)		Radical length (cm)		Germination index		Phytotoxicity (%)		Stress tolerance index	
	Untreated	Treated	Untreated	Treated	Untreated	Treated	Untreated	Treated	Untreated	Treated	Untreated	Treated
1	100 @ 0.00±	100 @ 0.00	00 @ 00 ±	00 @ 0.00	1.9 @ 0.04 ±	2.00 @ 0.01ˆa	0.95 @ 0.10±	1.0 @ 0.08ˆa	5.0 @ 0.01 ±	00 @ 00ˆns	0.95 @ 0.02±	1.00 @ 0.01ˆns
2.5	90 @ 0.00 ±	100 @ 0.00	10 @ 0.00 ±	00 @ 00	0.75 @ 0.02±	2.00 @ 0.02ˆa	0.33 @ 0.02±	1.0 @ 0.01ˆa	62.50 @ 2.77±	00 @ 00ˆa	0.37 @ 0.01±	1.00 @ 0.04ˆa
5.0	78 @ 0.00 ±	90 @ 0.00	22 @ 0.00 ±	10 @ 0.00	0.50 @ 0.04±	1.9 @ 0.02ˆa	0.19 @ 0.00±	0.85 @ 0.00ˆa	75 @ 2.98 ±	5.0 @ 1.4ˆa	0.25 @ 0.03±	0.95 @ 0.00ˆa
10	NG	85 @ 0.00	NG	15 @ 0.00	NG	1.1 @ 0.01ˆa	NG	0.46 @ 0.00ˆa	NG	45 @ 1.2ˆa	NG	0.55 @ 0.00ˆa
Control	100 @ 00 ±	100 @ 00	00 @ 00 ±	00 @ 00	2.00 @ 0.10±	2.00 @ 0.18ˆns	00 @ 00 ±	00 @ 00	00 @ 00 ±	00 @ 00	00 @ 00 ±	00 @ 00

*NG, no germination was observed; All the values are means of triplicate (*n* = 3) ± SD. The statistical significance between the values of untreated to their respective treated samples was evaluated by one-way ANOVA. ^a^Significant level *p* < 0.001. ^ns^Non-significant level *p* > 0.05. Control; tap water.*

### Genotoxicity Assay

**FIGURE 5:**
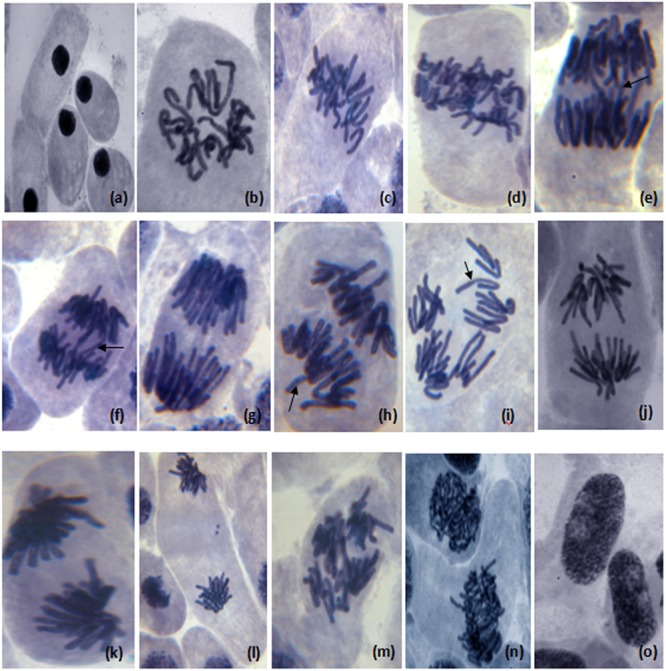
**Different chromosome aberration observed in meristematic cells of *Allium cepa* (2*n* = 16) treated with distillery sludge leachate; (a)** change in nucleus position in morphological altered cell, **(b)** prophase with genetic material loss, **(c,d)** disturb metaphase, **(e)** disturb anaphase, (c-mitosis) **(e,f)** chromosome bridge in anaphase, **(g)** sticky chromosome in anaphase, **(h,i)** laggard chromosome with diagonal anaphase, **(j)** disturb chromosome in anaphase, **(k)** sticky chromosome in anaphase, **(l,m)** sticky chromosome in telophase (chromosome adherence), **(n)** polyploidy cell, **(o)** apoptotic bodies (Magnification:1000×).

The results of genotoxic studies showed a concentration dependent reduction of the MI of root tip meristem cells of *Allium cepa*, after 24 h of treatment with different concentrations of distillery sludge leachate (**Table 4**). It was found that MI decreased with increasing concentrations (as percentages) of fresh sludge leachate compared to degraded sludge leachate and the decrease in MI was in the order 1% > 2.5% > 5% > 10%, which produced MI values of 16.6, 10.32, 6.68, 3.0, respectively. All tested samples induced chromosome abnormalities at all concentrations of fresh sludge leachate. Maximal chromosomal aberrations were recorded at a 10% concentration, showing 165% aberration and a total of 124 aberrant cells. The chromosomal aberrations observed included morphologically altered cells with losses of genetic material, disturbed metaphase, c-mitosis, chromosome bridges, sticky chromosomes, laggard chromosomes, polyploidy cells and apoptotic bodies (**Figure 5** and Supplementary Table 2). Exposure to distillery sludge leachate adversely affected the shape of cells of *A. cepa.* The percentage of morphologically altered cells was higher during treatment with different concentrations of leachate. Our study showed the presence of both c-mitosis (c-metaphase) and polyploid cells after treatment with distillery leachate exposure for 24 h. The frequency of cells with laggard and sticky chromosomes significantly increased with increasing distillery leachate concentrations (See Supplementary Table 2). These were seen mostly in anaphase-telophase aberration tests. In our study, the significant decrease in the MI and chromosome abnormalities observed after *in situ* bioremediation, indicated the detoxification of distillery leachate (**Table 4** and Supplementary Table 2). The most likely reason for the high genotoxicity and cytotoxicity of these industrial water samples is the complex assortment of organic pollutants produced during the sugarcane molasses distillation process. In summary, genotoxicity results indicate that distillery sludge and leachate produce genotoxicity and may exert potentially harmful effects on flora and fauna, however, this toxicity was reduced after 90 days of *in situ* bioremediation.

**Table 4:** Mitotic index and mitotic inhibition at different concentration of distillery sludge leachate.

PMDS leachate concentration (%)	Total no. of cells		Total no. of dividing cell		Mitotic index (%)		Mitotic inhibition (%)	
	Untreated	Treated	Untreated	Treated	Untreated	Treated	Untreated	Untreated
1.0	2500	2500	415 @ 20.75±	551 @ 19.28ˆa	16.6 @ 0.74 ±	22.04 @ 0.90ˆa	28.93 @ 1.27±	5.65 @ 0.19ˆa
2.5	2500	2500	258 @ 12.38±	475 @ 18.05ˆa	10.32 @ 0.36±	19.00 @ 0.86ˆa	55.82 @ 1.95±	18.66 @ 0.55ˆa
5.0	2500	2500	167 @ 8.14 ±	315 @ 35.12ˆa	6.68 @ 0.24 ±	12.6 @ 0.44ˆa	71.40 @ 3.28±	46.06 @ 1.42ˆa
10	2500	2500	75 @ 2.25 ±	269 @ 9.14ˆa	3.0 @ 0.12 ±	10.76 @ 0.40ˆa	87.15 @ 4.00±	53.93 @ 1.94ˆa
Control	2500	2500	584 @ 28.61±	584 @ 27.51ˆns	23.36 @ 0.93±	23.36 @ 1.14ˆns	–	–

*All the values are means of triplicate (*n* = 5) ± SD. The statistical significance between the values of untreated to their respective treated samples was evaluated by one-way ANOVA. ^a^Significant level *p* < 0.001. ^ns^Non-significant level *p* > 0.05. Control; tap water.*

## Discussion

Physicochemical analysis showed that sludge and leachate both have a high pH. This may be due to the presence of large amounts of salt, but the slight reduction in various physicochemical properties, as measured for leachate, was due to bioremediation by autochthonous bacterial communities; this alters the original chemical structure and properties of various organic constituents and salts in the sludge (Chandra and Kumar, 2017b). The alkalinity of the waste arises from the combined residual effect of carbonates, bicarbonates, and hydroxides, which are used in a distillery for pH adjustment during the fermentation process and the washing of fermentation products (Tiwari et al., 2013). While the EC of sludge and leachate was found to be 4.1 and 3.9, respectively, the high values of EC indicated the role of various cations and anions, such as sodium, chloride, nitrate, and ammonium ions, which are present in both fresh distillery sludge and leachate. The high pH and EC of sludge could be due to the presence of high concentrations of soluble salts. Several authors have also reported higher values of these cations and anions in distillery waste (Chandra et al., 2008; Chandra and Kumar, 2017b). Furthermore, in the present study the presence of various heavy metals along with melanoidins has also been shown. Heavy metals have a strong tendency to bind to melanoidins (Migo et al., 1997). The high content of heavy metals in distillery sludge could be due to the corrosive effect of sugarcane juice during the sugar manufacturing process. Metals may be added further during the fermentation and distillation processes of sugarcane molasses in distilleries, as it is finally discharged as a spent wash under highly acidic conditions and again, undergoes treatment in an anaerobic reactor and all of these processes potentially induce metal corrosion of metal pipes. This may be the main source of the heavy metal content of distillery sludge. The high concentrations of heavy metals and salts in PMDS are due to the condensation process that takes place during sugar manufacturing and alcohol production. Our findings corroborated well with earlier data (Chandra and Kumar, 2017b). The high levels of organic and inorganic parameters in PMDS are also concordant with previous reports (Chandra et al., 2008). The high content of several heavy metals is an environmental health hazard because they are known to be damaging to humans and animals due to their persistence and accumulation in the environment from contaminated sites. They are known to cause neural toxicity, renal disorders, asthma, and carcinogenic effects in humans and animals (Barakat, 2011). Therefore, the sludge has toxic properties and apparently does not degrade. There was a subsequent reduction in various physicochemical parameters of the distillery leachate after 90 days of *in situ* bioremediation; possibly due to the action of the microbial community resulting in the mineralization of complex compounds. The majority of bacteria, actinomycetes and fungi are saprophytes and so they decompose organic matter. They hydrolyze and oxidize various complex organic and inorganic compounds through enzymic processes, resulting in the reduction in some physicochemical parameters following *in situ* bioremediation.

Fourier transform-infrared spectrophotometry spectroscopy is an extensively used method for the determination of the functional groups present in organic compounds. The observed change in the peaks in FTIR spectra analysis of control and degraded sludge leachate suggests degradation of organic compounds has occurred after 90 days of bioremediation *in situ*. This indicated the conversion of complex toxic compounds into simpler, non-toxic molecules. The autochthonous bacterial communities employed during *in situ* remediation during the investigation potentially resulted in the cleavage of various chemical linkages.

Gas chromatography-mass spectrometry is a good technique for determining the organic pollutants in the environment. In our study, the majority of the organic compounds detected in distillery sludge and leachate posing a threat to the environment and human health are substances derived from biochemical processes at the various stages of sugar and alcohol production, and of effluent treatment. GC-MS analysis of organic extracts in the sample identified more than 35 organic compounds some of which were mutagens, carcinogens, and environmental endocrine disrupters. Octadecanoic acid and hexadecanoic were detected in our study, which have been reported to be anti-quorum sensing molecules in bacterial products (Singh et al., 2013). However, in some studies octadecanoic acid has also been reported to be a toxic compound in aquatic systems (Kamaya et al., 2003), while hexadecanoic acid has been reported to be a DNA fragmentation inducer in a human melanoma cell line (de Sousa Andrade et al., 2005). Similar compounds have been reported by various researchers, and so our data support results reported previously (González et al., 2000; Quinn et al., 2007). This finding also corroborated previous studies (Kaushik et al., 2010). These organic compounds constitute the main components of wastewater and discharge in sludge from yeast or original sugarcane molasses, which remains after the secondary treatment of distillery effluent. Apart from this, other compounds were also identified as stigmasta-5, 22-dien-3-ol(3 β,22E), stigmasterol and β-sitosterol. These are major phytosterols (plant sterol) with a chemical structure similar to cholesterol, soluble in water at all pH values and previously reported to be present in sugarcane (*Saccharum officinarum* L.) and the wastewater from sugarcane molasses. These compounds are screened under the list of environmental endocrine-disrupting chemicals (EDCs; as in USEPA, 2012). In a previous study, it was reported that in nature, aerobic, and/or anaerobic microorganisms may transform β-sitosterol and other sterols into androgenic hormones such as 5-β-androstan 3, 17-dione and androstan 4-en-3, 17-dion (Taylor et al., 1981). Such androstan derivatives of sterols may ultimately interfere with endocrine systems and produce hermaphroditism or other morphological defects. A role of these compounds in masculinizing the fish population and reducing fish numbers has been suggested (Jenkins et al., 2003). However, tetradecane and other similar phyo hydrocarbons (heptacosane dotriacontane, lanosta-8, 24 dien-3-one) have also been identified, and reported as key constituents of environmental pollutants responsible for dermal irritation (Muhammad et al., 2005). We have also detected several other organic compounds in control and degraded sludge leachates. This might occur as a residual fraction of the distillation process during ethanol production from fermented molasses slurry by yeast cells. However, the role of several other organic compounds detected in the environment is still of interest and requires detailed investigation for environmental safety.

Sequence analysis of the 16S rRNA genes of bacteria and archaea has been frequently used to characterize the taxonomic composition and phylogenetic diversity of environmental samples (Langille et al., 2013). In the present work, the RFLP technique was used to divulge phylogenetic relatedness among uncultured bacterial clones. RFLP analysis disclosed the presence of the major phyla *Bacillus*, followed by *Enterococcus*. The genus *Bacillus* is also characterized by its cellular properties, which are Gram-positive, have a low G+C content, are mostly straight rod shaped, and occur singly, in pairs, or chains. They also form endospores and grow aerobically. So, in the presence of organic compounds under anaerobic conditions they could grow slowly or are in the inactive phase and become viable after the discharge of sludge (Whitman, 2009). The heat resistant nature of the endospores accounts for the presence of *Bacillus* sp. in cooling tower water, as the heat-exchange process selects for heat resistant microorganisms that become even more dominant as water is recycled (Sharmin et al., 2013). Also, Park et al. (2007) reported the dominance of *Bacillus* sp. growing on a Rotating Activated *Bacillus* Contactor Biofilm (RABCS) as used for advanced wastewater treatment by culture-dependent methods. The second member of the phylum *Firmicutes* noted in this study was *Enterococcus* sp. These bacteria are characterized as being Gram-positive, cocci-shaped, often occurring in pairs (diplococci) or short chains, non-endospore forming, aerobic or facultative anaerobes, capable of the fermentation of sugars and of producing lactic acid or other acidic products. One study has also revealed the presence of *Bacillus* and *Enterococcus* sp. (España-Gamboa et al., 2012) in the anaerobic sludge of an upflow anaerobic sludge blanket (UASB) reactor for the treatment of distillery spent wash. This indicated that bacteria are able to exploit carbon compounds from spent wash as a necessary source of energy, and can adapt to this environment by using their endogenous enzymatic systems. This corroborates our results.

Based on sequence analyses recovered from the clone libraries, the phyla *Firmicutes* represented the dominant group of bacteria. PhyloChip analysis has also been used to assess the dominance of *Firmicutes* growing within sugarcane processing plants (Sharmin et al., 2013). Acharya et al. (2011) also studied the dynamics of the microbial communities within anaerobic biphasic fixed film bioreactor treatment distillery spent wash, showing the clear dominance by *Firmicutes* in a methanogenic bioreactor, which indicates the high degree of diversity of this phylum. The presence of *Firmicutes* as a dominant group is quite rare for natural samples. However, lignocellulosic material, such as sugarcane molasses/sugarcane bagasse represents an abundant, inexpensive source of organic material, which can be a carbon source for a growing bacterial population. Hence, the results of the current study agree with previously reported data from autochthonous bacterial communities in similar environments (Ch]andra et al., 2012). PMDS contains much more various organic and inorganic complex pollutants at much greater concentrations, but the autochthonous bacterial communities identified were specific and capable of growing in this environment.

The SG test is a common and basic tool used for toxicity evaluations of the environmental safety of industrial waste (OECD, 2003). A SG rate of 100% occurred with a 1% (v/v) concentration of fresh leachate and as the concentration increased, so the percent germination decreased. The promotion of seedling growth by lower concentrations of leachate might be due to the presence of lower concentrations of toxicant in samples tested. While the suppression of germination at high concentrations of leachate might be due to the presence of highly toxic organic compounds and dissolved solid, which were absorbed by the seeds before germination and affected various physiological and biochemical processes of SG (Bharagava and Chandra, 2010). Increases in the percentage germination in degraded sludge leachate may be due to the presence of less organic compounds, so creating a favorable environment for germination and utilization of nutrients present in the leachate prepared from the degraded sludge. Different concentrations of leachate and the different degrees of seed coat permeability led to different degrees of inhibition of germination. The inhibitory effect of the fresh sludge leachate on SG and seedling growth might be attributed to the high salt load and metal content, which induces both a high osmotic pressure and anaerobic conditions. It has also been reported that a high salt load and metal content acts as an inhibitor of plant hormones (amylases, auxins, gibberellins, and cytokinins), which are required mainly for SG, seedling growth and plant development, respectively (Ahsan et al., 2007). One study showed that distillery-sludge-amended soil delayed flowering and reduced pod formation in *P. mungo* L., which apparently provides evidence for the suppression of reproductive hormones by the toxicants present in distillery sludge (Chandra et al., 2008). These findings support the presence of high levels of EDCs plus other toxic compounds in distillery sludge.

Many national and international studies have focused on genotoxicity evaluation of industrial wastewater in different test models (Hemavanthi et al., 2015; Kumari et al., 2016). We selected the *A. cepa* assay due to its sensitivity and effectiveness in assessing effluent pollutants. The assay demonstrated that untreated samples were more toxic than the respective treated samples. The results of genotoxicity tests showed that MI decreased with increasing concentrations of fresh sludge leachate as compared to degraded sludge leachate. Trace metals and other organic pollutants have been considered responsible for diminishing the MI of *A. cepa* exposed to industrial wastewater (Chandra et al., 2005; Carita and Marin-Morales, 2008). A cytogenic effect was also observed that might be due to the presence of genotoxic compounds within the PMDS. We observed various chromosomal aberrations including morphologically altered cells with loss of genetic material, disturbed metaphase, c-mitosis, chromosome bridges, laggard chromosomes, sticky chromosomes, polyploidy cells, and apoptotic bodies. The reason for such effects could be due to the presence of toxic substances in the liquid medium, which may disturb division, causing a relatively high number of aberrations. c-Metaphase may result from the action of aneurogenic agents on the cell; compounds that promote complete inactivation of the mitotic spindle (Fiskesjo, 1993; Fernandes et al., 2007). Such alterations may generate other types of cell abnormalities, such as polyploid cells (Odeigah et al., 1997). The presence of chromosomal adherence reinforces the evidence of the aneugenic action of organic pollutants present in distillery leachate. However, organic pollutants disturb the balance in the quantity of histones or other proteins responsible for controlling the proper structure of nuclear chromatin. Chromosome stickiness reflects a highly toxic effect, which probably leads to cell death (Fiskesjo, 1997). Similar observations have been reported in *A. cepa* root after treatment with distillery effluent (Hemavanthi et al., 2015). This increased stickiness also leads to the formation of chromosome bridges. However, the frequency of cells with chromosome bridges significantly increased with increases in the concentration of distillery sludge leachate, indicating a clastogenic effect of leachate (Lerne and Marin-Morales, 2009) involving one or more chromosomes. Chromosome bridges were observed here, and may have occurred due to the misrepair of DNA, telomere end fusions or even from chromosome adherence. Our findings are well corroborated with earlier studies (Hemavanthi et al., 2015).

## Conclusion

This study has revealed that PMDS and its leachate contain several residual recalcitrant organic pollutants plus heavy metals, the majority of which are environmental toxicants (i.e., octadecanoic acid), endocrine disrupting chemicals (i.e., β-sitosterol) and also DNA fragmentation inducers (i.e., tetradecanoic acid), which are still fairly unknown. During bioremediation of PMDS *in situ, Bacillus* sp., and *Enterococcus* sp., were noted growing dominantly as autochthonous bacterial communities of the phylum *Firmicutes.* This showed a capability to degrade the toxic pollutants present in PMDS. This bacterial community also demonstrated a special niche in high concentrations of Fe, Zn, Cu, Mn, and Pb, and an environment rich in complex organic compounds containing mutagenic molecules and EDCs. The findings of the present study will be useful for monitoring and managing distillery waste environmentally and for the eco-restoration of polluted sites.

## Author Contributions

RC did leading role for designing of experiments, analysis of pollutants, and bacterial community. While, VK played role as team member and supported in experimental work and data arrangement. Further, the co-author also supported to corresponding author, i.e., RC for manuscript formatting and phytotoxicity and genotoxicity assay and graph preparation. Thus, both authors justified their role for manuscript preparation and communication.

## Conflict of Interest Statement

The authors declare that the research was conducted in the absence of any commercial or financial relationships that could be construed as a potential conflict of interest.

## Footnotes

1 http://ncbi.nlm.nih.gov/
