# Supplementary material for: Detection of Androgenic-Mutagenic Compounds and Potential Autochthonous Bacterial Communities during In Situ Bioremediation of Post-methanated Distillery Sludge
Source: Front Microbiol. 2017 May 17;8:887. doi: 10.3389/fmicb.2017.00887 (PMC5434103; doi:10.3389/fmicb.2017.00887)
Supplement: Supplementary file 1 [file Data_Sheet_1.doc]

**Supplementary Material**

**Detection of Androgenic-Mutagenic Compounds and Potential Autochthonous Bacterial Communities During In-Situ Bioremediation of Post Methanated Distillery Sludge**

**Ram Chandra* and Vineet Kumar**

Department of Environmental Microbiology, School for Environmental Sciences, Babasaheb Bhimrao Ambedkar Central University, Vidya Vihar, Raebareli Road, Lucknow,

Uttar Pradesh -226025, India

Corresponding author: [rc_microitrc@yahoo.co.in](mailto:rc_microitrc@yahoo.co.in); prof.chandrabbau@gmail.com

1. **Supplementary Figures and Tables**

**1.1 Supplementary Figures**

**
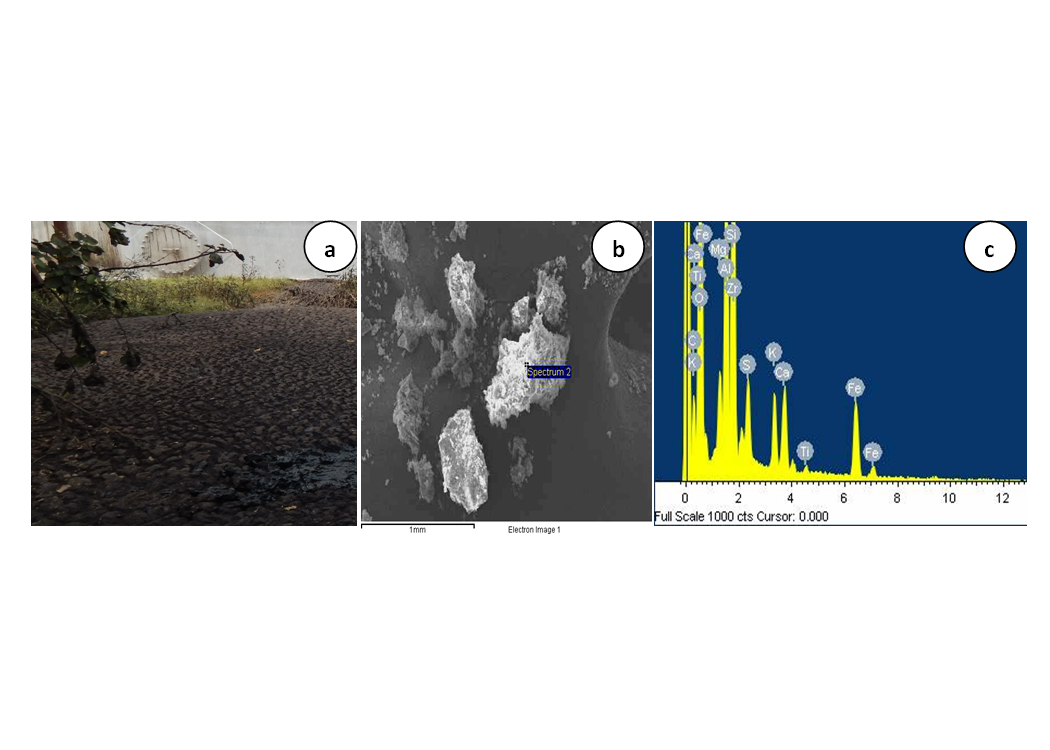
**

**Supplementary Figure 1.** Post methanated distillery sludge; (a) distillery sludge dumped after methanogenesis of spent wash (b) SEM image of the morphology of sludge (100×) (c) elemental analysis image (EDS) of sludge


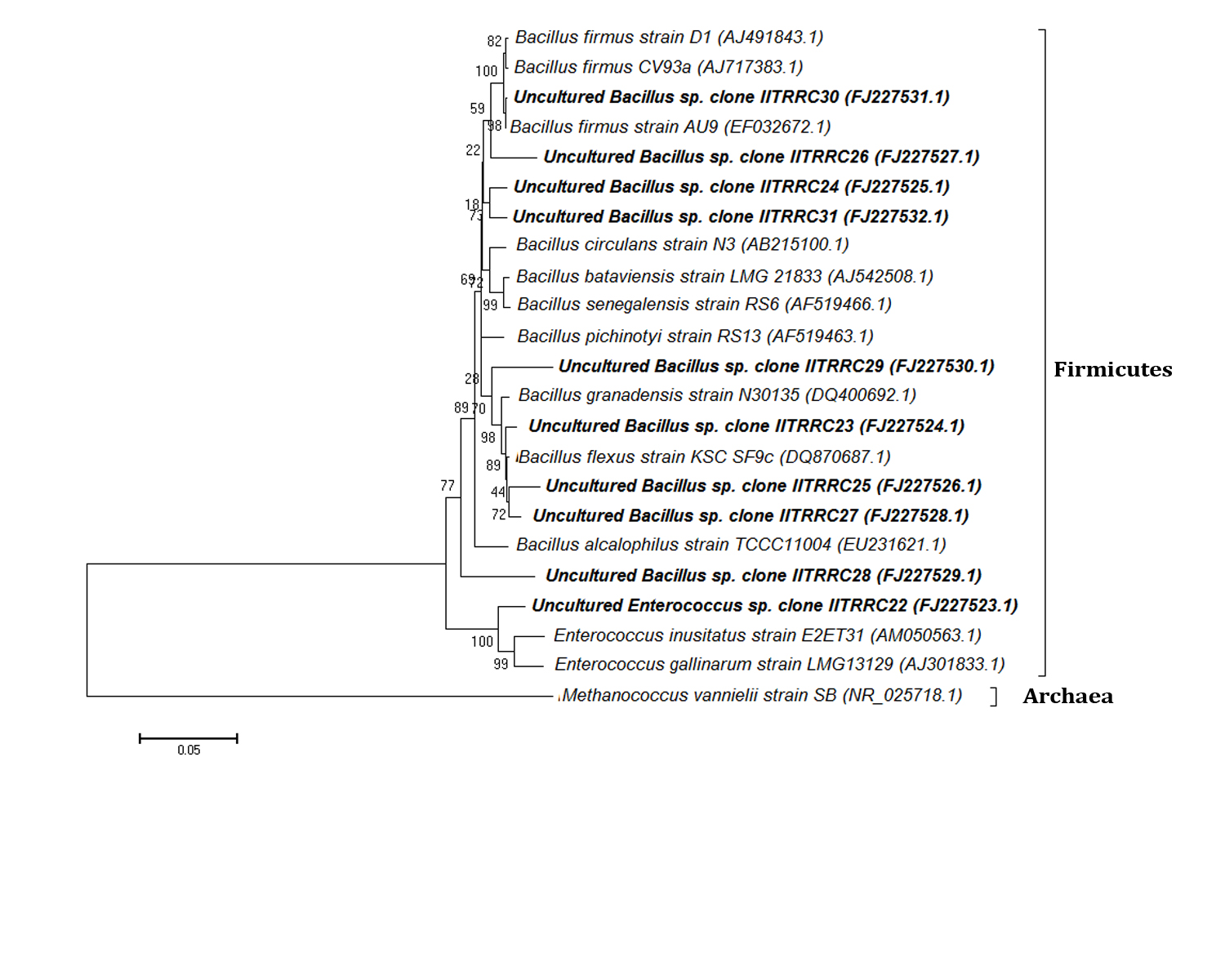


**Supplementary Figure 2.** Phylogenetic tree generated via Neighbour-Joining method showing the relationship of uncultured bacterial community growing in PMDS with their closest homologues based on 16S rRNA gene sequences. The query sequences were denoted in boldface, after tree reconstruction it showed, that all the query sequences were lying in phylum *Firmicutes* of bacteria. All the accession numbers of sequences were written in square bracket at the end of sequence name. *Methanococcus vannielii* strain SB in the Archaea domain was utilised as the out group. The scale bar represents 0.05 changes per nucleotide.

**1.1 Supplementary Table**

**Supplementary Table 1.** Elemental composition (EDS) of PMDS

| Element | Weight % |
| --- | --- |
| C-K | 24.10 |
| O-K | 50.08 |
| Mg-K | 1.49 |
| Al-K | 5.22 |
| Si-K | 8.67 |
| S-K | 1.41 |
| K-K | 1.55 |
| Ca-K | 1.81 |
| Ti-K | 0.20 |
| Fe-K | 4.27 |
| Zr-K | 1.19 |
| Total | 100.00 |

The K following every element indicated the K-shell of the specific atom

**Supplementary Table 2.** Cytological effect of distiller leachate on the *Allium cepa* root meristem cell after 24 hrs

| Aberrant  Leachate  concentration | Morphological alerted cell | Prolonged prophase | Disturb metaphase | Multipolar anaphase | C-mitosis  (vagrant chromosome) | Chromosome bridge | Spindle disturbance cell at anaphase | Laggard chromosome multipolarity cell | Sticky chromosome | Polyploid cell | Apoptotic bodies | % Aberrant  cells |
| --- | --- | --- | --- | --- | --- | --- | --- | --- | --- | --- | --- | --- |
| Untreated | | | | | | | | | | | |  |
| 0 | 0 | 1 | 0 | 0 | 1 | 0 | 0 | 0 | 1 | 0 | 0 | 0.51 |
| 1 | 0 | 1 | 2 | 0 | 2 | 1 | 2 | 1 | 3 | 2 | 1 | 3.61 |
| 2.5 | 20 | 10 | 8 | 3 | 8 | 6 | 4 | 2 | 5 | 10 | 1 | 29.84 |
| 5.0 | 50 | 17 | 12 | 5 | 10 | 9 | 7 | 4 | 11 | 12 | 2 | 85.62 |
| 10 | 42 | 11 | 15 | 2 | 12 | 11 | 9 | 7 | 12 | 2 | 1 | 165 |
| Treated | | | | | | | | | | | |  |
| 0 | 0 | 1 | 0 | 0 | 1 | 0 | 0 | 0 | 1 | 0 | 0 | 0.51 |
| 1 | 0 | 0 | 0 | 0 | 0 | 0 | 0 | 0 | 0 | 0 | 0 | 0.00 |
| 2.5 | 2 | 0 | 0 | 0 | 0 | 0 | 0 | 0 | 0 | 0 | 0 | 0.42 |
| 5.0 | 3 | 3 | 3 | 1 | 0 | 1 | 0 | 1 | 2 | 1 | 1 | 5.07 |
| 10 | 5 | 4 | 2 | 1 | 2 | 1 | 1 | 1 | 1 | 0 | 1 | 7.06 |
